# Supplementary material for: A prospective survey of Streptococcus pyogenes infections in French Brittany from 2009 to 2017: Comprehensive dynamic of new emergent emm genotypes
Source: PLoS One. 2020 Dec 17;15(12):e0244063. doi: 10.1371/journal.pone.0244063 (PMC7746304; doi:10.1371/journal.pone.0244063)

**S1 Fig. Geographical distribution of collected cases in French Brittany according to the residence of patients**


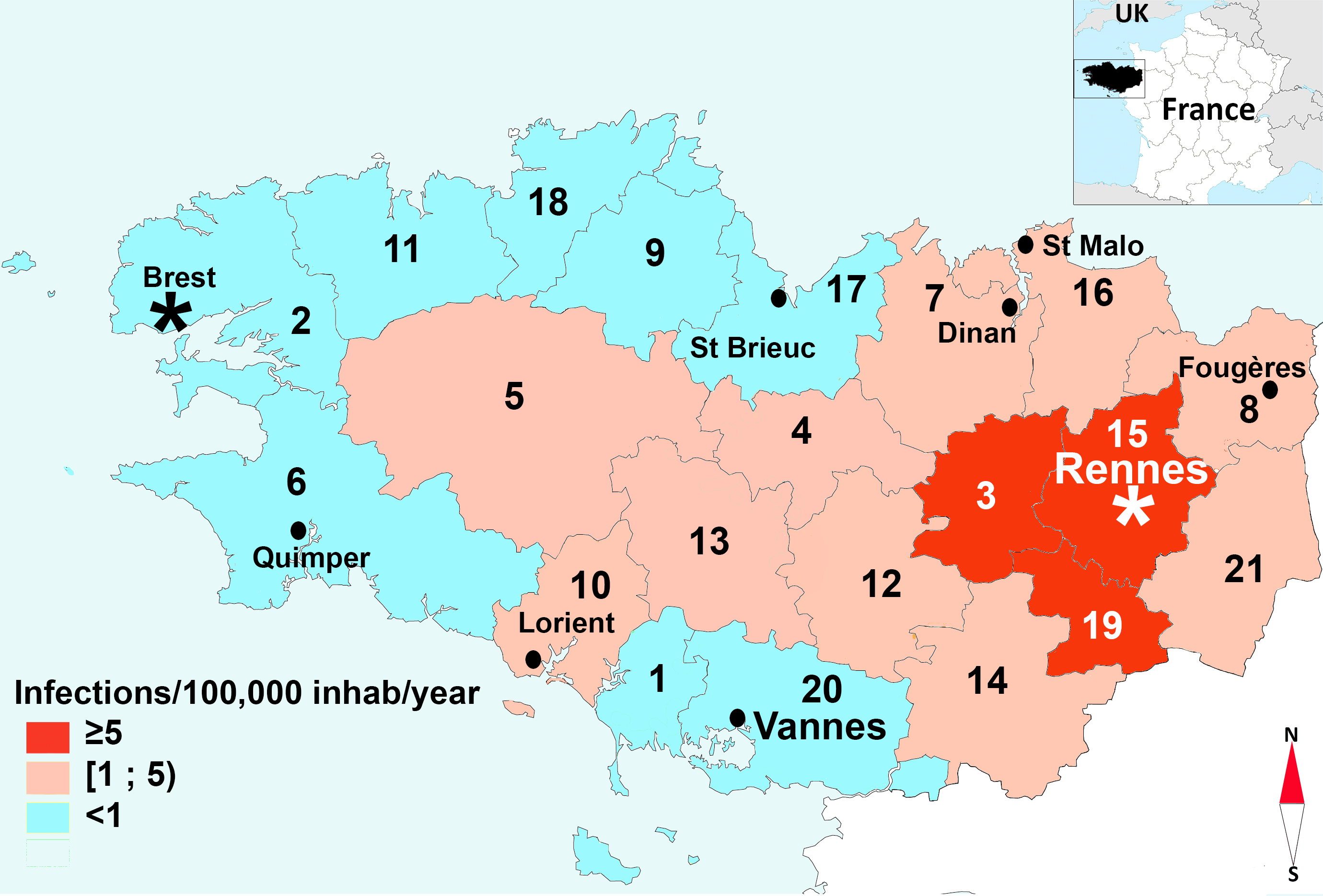

Supplement: S1 Fig — The reported numbers corresponded to the 21 areas of French Brittany: 1) Auray; 2) Brest; 3) Broceliande; 4) Centre Bretagne; 5) COB; 6) Cornouaille; 7) Dinan; 8) Fougères; 9) Guingamp; 10) Lorient; 11) Morlaix; 12) Ploermel; 13) Pontivy; 14) Redon; 15) Rennes; 16) Saint-Malo; 17) Saint Brieuc; 18) Tregor-Goelo; 19) Vallons-Vilaine; 20) Vannes; 21) Vitré. Regional hospitals (*) and University Hospital Centres (•) were indicated on the map. For each area, the average number of cases collected/100,000 inhabitants/year were reported according to the colour legend. (DOCX) [file pone.0244063.s001.docx]
