# Supplementary figures and images for: A prospective survey of Streptococcus pyogenes infections in French Brittany from 2009 to 2017: Comprehensive dynamic of new emergent emm genotypes
Source: PLoS One. 2020 Dec 17;15(12):e0244063. doi: 10.1371/journal.pone.0244063 (PMC7746304; doi:10.1371/journal.pone.0244063)

**S2 Fig. Seasonal variation of infection rates.**


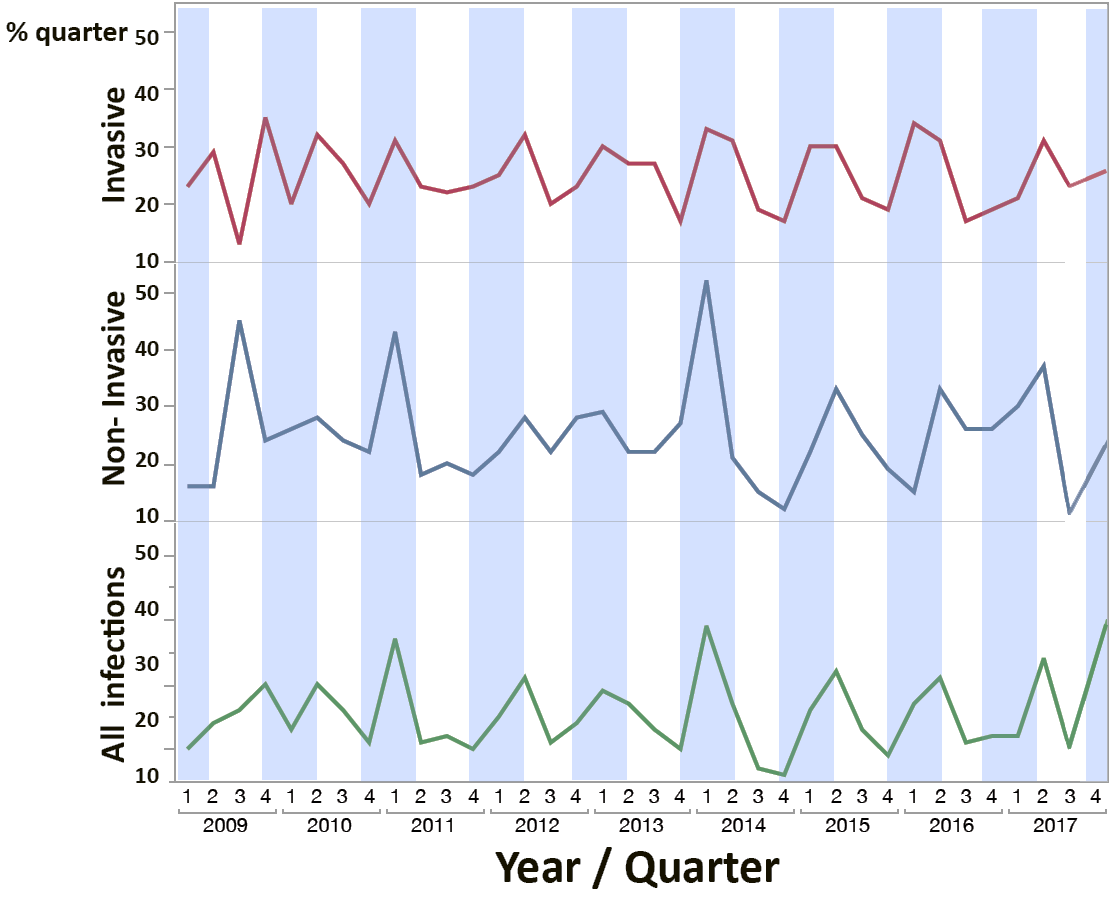

Supplement: S2 Fig — All infections (green), non-invasive (blue), and invasive infections (red) were broken down by year, and rates of infections were given for each quarter. 1: January to March; 2: April to June; 3: July to September; 4: October to December. (DOCX) [file pone.0244063.s002.docx]
