# Supplementary material for: A prospective survey of Streptococcus pyogenes infections in French Brittany from 2009 to 2017: Comprehensive dynamic of new emergent emm genotypes
Source: PLoS One. 2020 Dec 17;15(12):e0244063. doi: 10.1371/journal.pone.0244063 (PMC7746304; doi:10.1371/journal.pone.0244063)
Supplement: S3 Fig — The rates for males, invasive infections and the portal of entry were indicated for each age group. ENT-Resp = Ear Nose and Throat and Respiratory. *n = 889 infections/942 collected cases (49 carriage and 4 cases with missing values were not included). (DOCX) [file pone.0244063.s003.docx]

**S3 Fig. Age group distribution of GAS infections**


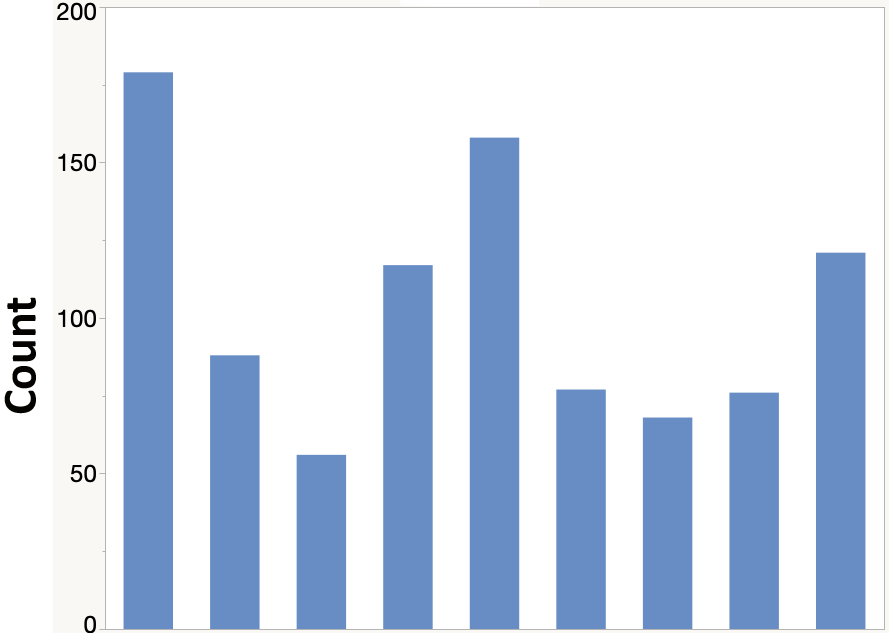


| **Age categories** | [0-5) | [5- 10) | [10-20) | [20-30) | [30-40) | [40 50) | [50-60) | [60-70) | ≥70 | Total | *p-*value |
| --- | --- | --- | --- | --- | --- | --- | --- | --- | --- | --- | --- |
| Infections cases | 166 | 85 | 56 | 107 | 151 | 76 | 63 | 71 | 114 | 889* |  |
| Males n (%) | 96 (57) | 44 (52) | 27 (48) | 56 (52) | 69 (46) | 53 (70) | 37 (59) | 38 (54) | 5 (50) | 477 | 0.0583 |
| Invasive infect. / Total infect. (%) | 76/166 (46) | 38/85 (45) | 37/56 (66) | 58/107 (54) | 96/151 (64) | 52/76 (68) | 40/63 (63) | 49/71 (69) | 93/114 (82) | 539 | <0.0001 |
| **Portal of entry**  <0.0001 | | | | | | | | | | | |
| Cutaneous n (%) | 67 (40) | 38 (45) | 31 (55) | 50 (47) | 71 (47) | 52 (68) | 42 (67) | 49 (69) | 91 (80) | 491 |  |
| ENT-Resp n (%) | 55 (33) | 27 (31) | 20 (36) | 32 (30) | 43 (28) | 13 (17) | 11 (17) | 5 (7) | 17(15) | 223 |  |
| Anogenital n (%) | 42 (25) | 19 (22) | 3 (5) | 24 (22) | 31 (20) | 6 (8) | 7 (11) | 11 (15) | 1 (1) | 144 |  |
| Not Kown n (%) | 2 (1.2) | 1 (1) | 2 (4) | 1 (1) | 6 (4) | 5 (7) | 4 (6) | 6 (8) | 5 (4) | 31 |  |
