# Supplementary material for: A prospective survey of Streptococcus pyogenes infections in French Brittany from 2009 to 2017: Comprehensive dynamic of new emergent emm genotypes
Source: PLoS One. 2020 Dec 17;15(12):e0244063. doi: 10.1371/journal.pone.0244063 (PMC7746304; doi:10.1371/journal.pone.0244063)
Supplement: S1 Table — For each identified emm types, the total number of GAS isolates (n) and percentage of the total (%) were indicated in the corresponding column. For the most frequent genotypes (n > 10 isolates) we performed a categorical analysis (Fisher’s exact test) to compare the rate of their occurrence “In Rennes” vs “Out Rennes” groups. Simpson’s Indexes of Diversity (SDI) and their comparison were given at the bottom of the table. * Among the 942 emm-typed GAS isolates, 1 missed value for the residential area. (DOCX) [file pone.0244063.s004.docx]

**S1 Table:** ***Emm* types diversity “In Rennes” area and “Out Rennes”**

| ***emm* types** | **Total**  **(942)***  **n (%)** | **“In Rennes”**  **(453)**  **n (%)** | **“Out Rennes”**  **(488)**  **n (%)** | ***p*-value** |
| --- | --- | --- | --- | --- |
| 28 | 147 (15.61) | 57 (6.06) | 90 (9.56) | **0.0151** |
| 89 | 142 (15.07) | 60 (6.38) | 81 (8.61) | 0.1703 |
| 1 | 136 (14.44) | 60 (6.38) | 76 (8.08) | 0.3536 |
| 4 | 75 (7.96) | 34 (3.61) | 41 (4.36) | 0.6319 |
| 12 | 63 (6.69) | 28 (2.98) | 35 (3.72) | 0.6024 |
| 3 | 38 (4.03) | 20 (2.13) | 18 (1.91) | 0.6211 |
| 6 | 37 (3.93) | 20 (2.13) | 17 (1.81) | 0.5046 |
| 77 | 35 (3.72) | 14 (1.49) | 21 (2.23) | 0.3898 |
| 44 | 32 (3.4) | 22 (2.34) | 10 (1.06) | **0.0193** |
| 83 | 20 (2.12) | 7 (0.74) | 13 (1.38) | 0.2648 |
| 75 | 48 (5.1) | 27 (2.87) | 21 (2.23) | 0.2995 |
| 66 | 14 (1.49) | 13 (1.38) | 1 (0.11) | **0.0006** |
| 87 | 26 (2.76) | 17 (1.81) | 9 (0.96) | 0.1097 |
| 2 | 13 (1.38) | 4 (0.43) | 9 (0.96) | 0.2679 |
| 11 | 8 (0.85) | 5 (0.53) | 3 (0.32) | - |
| 81 | 8 (0.85) | 1 (0.11) | 7 (0.74) | - |
| 86 | 6 (0.64) | 5 (0.53) | 1 (0.11) | - |
| 63 | 5 (0.53) | 2 (0.21) | 3 (0.32) | - |
| 108 | 5 (0.53) | 5 (0.53) | 0 (0) | - |
| 82 | 5 (0.53) | 2 (0.21) | 3 (0.32) | - |
| 9 | 4 (0.42) | 2 (0.21) | 2 (0.21) | - |
| 22 | 4 (0.42) | 2 (0.21) | 2 (0.21) | - |
| 88 | 4 (0.42) | 0 (0) | 4 (0.43) | - |
| 92 | 4 (0.42) | 2 (0.21) | 2 (0.21) | - |
| 102 | 4 (0.42) | 2 (0.21) | 2 (0.21) | - |
| 5 | 3 (0.32) | 2 (0.21) | 1 (0.11) | - |
| 53 | 3 (0.32) | 3 (0.32) | 0 (0) | - |
| 58 | 3 (0.32) | 2 (0.21) | 1 (0.11) | - |
| 76 | 3 (0.32) | 2 (0.21) | 1 (0.11) | - |
| 95 | 3 (0.32) | 2 (0.21) | 1 (0.11) | - |
| 100 | 3 (0.32) | 3 (0.32) | 0 (0) | - |
| 118 | 3 (0.32) | 2 (0.21) | 1 (0.11) | - |
| 169 | 3 (0.32) | 2 (0.21) | 1 (0.11) | - |
| 8 | 2 (0.21) | 0 (0) | 2 (0.21) | - |
| 49 | 2 (0.21) | 2 (0.21) | 0 (0) | - |
| 73 | 2 (0.21) | 1 (0.11) | 1 (0.11) | - |
| 80 | 2 (0.21) | 1 (0.11) | 1 (0.11) | - |
| 94 | 2 (0.21) | 1 (0.11) | 1 (0.11) | - |
| 110 | 2 (0.21) | 2 (0.21) | 0 (0) | - |
| 103 | 2 (0.21) | 2 (0.21) | 0 (0) | - |
| 13 | 1 (0.11) | 1 (0.11) | 0 (0) | - |
| 25 | 1 (0.11) | 0 (0) | 1 (0.11) | - |
| 29 | 1 (0.11) | 1 (0.11) | 0 (0) | - |
| 32 | 1 (0.11) | 1 (0.11) | 0 (0) | - |
| 42 | 1 (0.11) | 0 (0) | 1 (0.11) | - |
| 48 | 1 (0.11) | 1 (0.11) | 0 (0) | - |
| 54 | 1 (0.11) | 1 (0.11) | 0 (0) | - |
| 60 | 1 (0.11) | 1 (0.11) | 0 (0) | - |
| 65 | 1 (0.11) | 0 (0) | 1 (0.11) | - |
| 68 | 1 (0.11) | 1 (0.11) | 0 (0) | - |
| 85 | 1 (0.11) | 0 (0) | 1 (0.11) | - |
| 72 | 1 (0.11) | 0 (0) | 1 (0.11) | - |
| 90 | 1 (0.11) | 1 (0.11) | 0 (0) | - |
| 104 | 1 (0.11) | 1 (0.11) | 0 (0) | - |
| 105 | 1 (0.11) | 1 (0.11) | 0 (0) | - |
| 112 | 1 (0.11) | 1 (0.11) | 0 (0) | - |
| 116 | 1 (0.11) | 1 (0.11) | 0 (0) | - |
| 121 | 1 (0.11) | 0 (0) | 1 (0.11) | - |
| 180 | 1 (0.11) | 1 (0.11) | 0 (0) | - |
| 230 | 1 (0.11) | 1 (0.11) | 0 (0) | - |
| 183 | 1 (0.11) | 1 (0.11) | 0 (0) |  |

| SDI    CI(95%) | 0.851  (0.779-0.922) | 0.831  (0.777-0.891) | 0.803  (0.732-0.873) |
| --- | --- | --- | --- |

| **Comparision SDI** | p-values | |  |  |
| --- | --- | --- | --- | --- |
| Overall vs"In Rennes" | 0.419 | |  |  |
| Overall vs"Out- Rennes" | 0.362 | |  |  |
| "In Rennes" vs "Out-Rennes | 0.320 | |  |  |
|  | | |  | |
