# Supplementary material for: A prospective survey of Streptococcus pyogenes infections in French Brittany from 2009 to 2017: Comprehensive dynamic of new emergent emm genotypes
Source: PLoS One. 2020 Dec 17;15(12):e0244063. doi: 10.1371/journal.pone.0244063 (PMC7746304; doi:10.1371/journal.pone.0244063)
Supplement: Attachment — Submitted filename: Responses to the editor.docx [file pone.0244063.s007.docx]

**Response and modifications to each point raised by the academic editor and reviewer(s)**

Issues for the authors’ consideration:

References in abstract (line 37). Please delete.

The references have been deleted. Then all the references have been reordered accordingly.

Lines 73-75, “In contrast, in Africa and the Pacific region, the distribution of emm types exhibits a higher diversity explained by the non-observed dominant emm types [13].” Use of English.

The sentence has been changed by the following:

“In contrast, in Africa and the Pacific Islands, the distribution of emm-types is more diverse and does not show dominant emm-types.”

Lines 79-80, “In tropical countries, the most circulating emm types of GAS are of emm pattern D (skin tropism) or E (both skin and pharyngeal tropism) (…)”. Use of English.

The sentence has been changed by the following:

“In tropical countries, the most frequently isolated *emm* types of GAS belong to *emm* pattern D (skin tropism) or E (no specific tropism), as opposed to temperate regions where there are more strains of *emm* pattern A–C (pharyngeal tropism).”

Lines 169-170, “Once validated, the dataset basis was completely anonymized.” The authors probably mean that the database was completely anonymized.

“dataset basis” has been changed to “database”

Lines 177-179, “Out of the 21 areas of French Brittany, and as expected, GAS isolates were predominantly recovered from patients living in Rennes and surrounding areas and then decreased gradually (S1 Fig)”. Ambiguous meaning.

The sentence has been changed by the following:

“ The diagnosis of GAS infections was mainly performed within the UHC of Rennes, and explain that, over the 21 regions of French Brittany, the majority of GAS isolates was recovered from patients residing in Rennes and the neighbouring areas (Fig S1).

Lines 180-182, “During the surveillance period, and as previously described, we also observed seasonal variation of the rate of infections (invasive and non-invasive), culminating in autumn/winter (S2 Fig)[23].” Perhaps “peaking”?

“culminating” has been changed to “peaking”

Lines 231-232, “(…) the final diagnosis was septic arthritis (n=14), isolated bacteraemia (12), central nervous system infections (2), primary peritonitis (2), and pericarditis (1).” Here and elsewhere if number of cases is meant then these should be indicated by n=.

For all parentheses indicating the number of cases, an "n =" has been added

Lines 358-361, “Finally, emm types clustered as a single protein, and for which it has been proposed that their M protein could have different immunological, structural, and functional characteristics were grouped in the same raw and encompassed “Prevalent” (emm6) and “Sporadic” (emm5, emm29, and emm105) emm types (Fig 3).” Replace raw with row?

“raw” has been replaced with “row”

Lines 368-369, “Deciphering the temporal dynamic of emm genotypes, we observed five “Emergent” emm types that belonged to emm-clusters that “Sporadic” but not “Prevalent” genotypes were members of.” Use of English.

The sentence has been changed by the following:

“Deciphering the temporal dynamics of the *emm* genotypes in our studied population, we observed that the five "Emergent" *emm* types never belonged to clusters within which "Prevalent" genotypes have been identified.”

Lines 436-437, “(…)(skin or generalist rather throat specialist  strains)(…)” missing “than”.

“than” has been added

Lines 456-457, “This genotype upsurges last decades and has been linked recently to the emergence(…)”. Use of English.

The sentence has been changed as follows:

” This genotype upsurges last decades and has recently been associated with the emergence of a new successful clade variant that has undergone several genetic modifications affecting known virulence factors.”
